# Arbitrary cross-section SEM-cathodoluminescence imaging of growth sectors and local carrier concentrations within micro-sampled semiconductor nanorods

**DOI:** 10.1038/ncomms10609

**Published:** 2016-02-16

**Authors:** Kentaro Watanabe, Takahiro Nagata, Seungjun Oh, Yutaka Wakayama, Takashi Sekiguchi, János Volk, Yoshiaki Nakamura

**Affiliations:** 1 International Center for Materials Nanoarchitectonics, National Institute for Materials Science, 1-1 Namiki, Ibaraki 305-0044, Japan; 2 Graduate School of Engineering Science, Osaka University, 1-3 Machikaneyama-cho, Osaka 560-8531, Japan; 3 MTA EK Institute of Technical Physics and Materials Science, Konkoly Thege M. ut 29-33, Budapest 1121, Hungary

## Abstract

Future one-dimensional electronics require single-crystalline semiconductor free-standing nanorods grown with uniform electrical properties. However, this is currently unrealistic as each crystallographic plane of a nanorod grows at unique incorporation rates of environmental dopants, which forms axial and lateral growth sectors with different carrier concentrations. Here we propose a series of techniques that micro-sample a free-standing nanorod of interest, fabricate its arbitrary cross-sections by controlling focused ion beam incidence orientation, and visualize its internal carrier concentration map. ZnO nanorods are grown by selective area homoepitaxy in precursor aqueous solution, each of which has a (0001):+*c* top-plane and six {1–100}:*m* side-planes. Near-band-edge cathodoluminescence nanospectroscopy evaluates carrier concentration map within a nanorod at high spatial resolution (60 nm) and high sensitivity. It also visualizes *+c* and *m* growth sectors at arbitrary nanorod cross-section and history of local transient growth events within each growth sector. Our technique paves the way for well-defined bottom-up nanoelectronics.

## Abstract (web summary)

Semiconductor nanocrystals are potential nanoelectronic materials but often display nonuniform electric properties due to their anisotropic growths. Here, the authors report cross-sectional cathodoluminescence imaging of a single-crystalline ZnO nanowire to resolve its growth sectors with different carrier concentrations.

## Graphical abstract

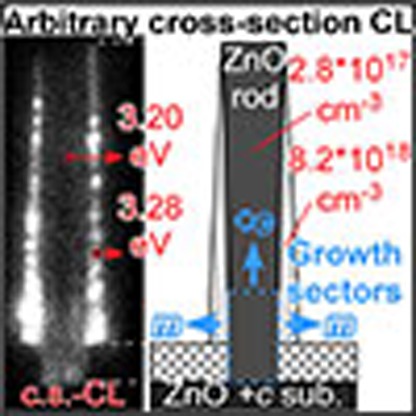

Bottom-up growths (for example, wet chemical growth^1^,^2^ and molecular beam epitaxy^3^,^4^) of semiconductor nanocrystals are paid attention because of the limitation of semiconductor microfabrication. Especially, a free-standing nanowire or nanorod has several exotic properties applicable to unique one-dimensional electronics; high flexibility (large fracture strain) for strain-engineered ultrafast nanowire field-effect transistors^5^ and for efficient nanopiezotronic devices^6^, large surface-to-volume ratio for sensitive solar cells^7^ and gas sensors^8^, and wave-guides for Fabry-Perot nanolasers^9^,^10^,^11^. A nanowire with uniform electric properties besides uniform diameter is essential for one-dimensional electronics with high device designability and reproducibility, which should be driven by well-defined current density.

Up to now, electrical properties of a bottom-up nanocrystal and its device properties are reported assuming electrical uniformities. However, this is idealistic because each crystallographic plane surface has an atomic arrangement with unique chemical activity (for example, etching rate^12^,^13^, host crystal growth rate^14^,^15^ and incorporation rates of point-defects), as is reported on plane-dependent donor concentration in ZnO bulk crystal^13^,^16^ and amphoteric Si-doping in GaAs substrates^17^,^18^. Thus, a nanocrystal grown in multiple crystallographic orientations has corresponding growth sectors with different electric properties, which is critical for above electronic applications. Further, growth sectors within a nanocrystal are not fully described macroscopically by pure crystallographic planes at constant growth rates^19^,^20^,^21^,^22^,^23^. (For example, a free-standing nanowire with uniform diameter is also idealistic^24^.) In reality, a growth form of a nanocrystal may evolve with growth duration depending on the local and temporal growth environment on each crystallographic plane, where spontaneous surface roughening or new plane formation may take place. Thus, it is difficult to foresee the internal spatial distributions of growth sectors. We demand a novel experimental technique which reveals local electrical properties within a nanocrystal, especially those due to the growth sectors, at high spatial resolution and high sensitivity.

A recent report on nanowire Hall effect measurement reveals carrier transport properties of an entire free-standing nanowire between micro-fabricated electrical contacts^25^. However, there is no adequate technique that can probe such electrical non-uniformities within a free-standing nanocrystal at sufficient sensitivity. Atom probe tomography^26^,^27^ and cross-sectional scanning transmission electron microscopy^28^ may visualize dopant species at atomic resolutions. However, none of them has sufficient sensitivities to probe local concentration and investigate electrical activity of point-defects within nominally undoped semiconductors. Cross-sectional scanning tunnelling microscopy^29^ may visualize electrical activities of dopants at sub-surfaces at atomic resolution. However, atomically smooth specimen cleavage is required to obtain noticeable contrasts of impurity atoms or vacancies, which is not available for a free-standing nanocrystals of interest up to now.

Contrary, cathodoluminescence (CL) nanospectroscopy is promising for its high sensitivity (∼10^15^ cm^−3^) of shallow level point-defects and nanometric resolution achievable by low-energy electron beam (e-beam) probe^30^,^31^,^32^,^33^,^34^. Cross-sectional CL imaging of growth sector interface is reported on hydrothermally grown ZnO bulk crystals with (0001):+*c*, {1–100}:*m* and {1–101}:*+p* planes^35^ and on hydride vapour phase epitaxy grown bulk GaN *+c* films containing hexagonal micro-pits with *+p* planes^36^,^37^. Plane-dependent CL behaviours of ZnO nanorod are reported recently^38^. However, cross-sectional CL technique is not applied to any semiconductor free-standing nanocrystal up to now.

ZnO single-crystalline free-standing nanorod array is chosen to demonstrate our technique, as it is suitable for low-cost optoelectronics and piezoelectronics. Wurzite ZnO is a piezoelectric wide-gap semiconductor with direct bandgap (*E*_g_=3.37 eV), a large exciton binding energy (*E*_b_=60 meV)^39^ and a large piezoelectric coefficient along its polar *c*-axis (*d*_cc_=12.4 pm V^−1^)^40^, which can be synthesized at low temperature and at atmospheric pressure in precursor aqueous solution: by simple chemical synthesis^41^,^42^,^43^,^44^,^45^,^46^,^47^,^48^,^49^,^50^,^51^,^52^,^53^,^54^ or by electrodeposition^55^,^56^,^57^,^58^.

Here we demonstrate a series of techniques to micro-sample a free-standing nanocrystal of interest and to visualize its internal local carrier concentration, especially those due to growth sectors, from arbitrary cross-sectional orientations. Circular growth windows are fabricated by e-beam lithography in a polymethyl methacrylate (PMMA) e-beam-resist film spin-coated on a single-crystalline ZnO (0001) substrate. ZnO single-crystalline free-standing nanorod arrays are then homoepitaxially grown at a time in precursor aqueous solution from circular growth window arrays with different diameters (*D*_w_; Supplementary Fig. 1). Scanning electron microscopy (SEM) observation shows that any nanorod grows from each circular growth window to have a +*c* top-plane and six *m* side-planes (Supplementary Figs 2 and 3). A nanorod is micro-sampled and its cross-section is fabricated by controlling crystallographic orientation of focused ion beam (FIB) incidence. Cross-section CL imaging of the nanorod visualizes *+c* and *m* growth sectors within an entire nanorod at high spatial resolution (<60 nm). CL nanospectroscopy on *+c* and *m* planes reveals their local carrier concentration differences and its quantitative accuracy is confirmed by ‘differential' *I–V* measurements of an individual ZnO free-standing nanorod (Supplementary Fig. 4).

## Results

### Room temperature CL nanospectroscopy of ZnO nanorods

**Figure 1:**
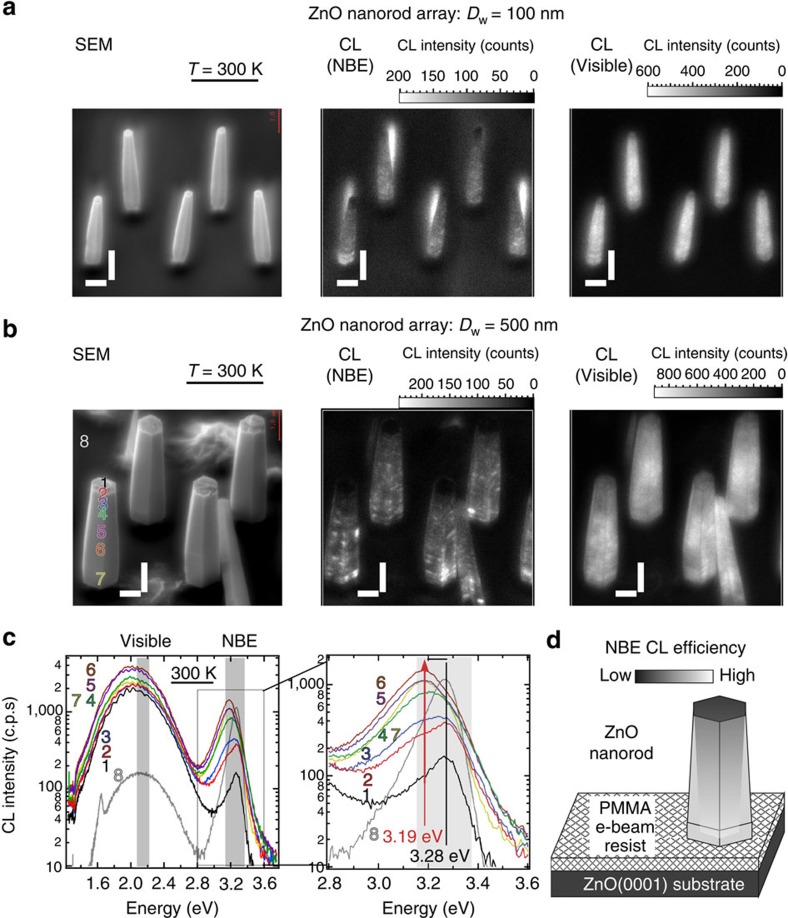
Bird's-eye view NBE/Visible CL imaging of ZnO free-standing nanorods. SEM and monochromatic (NBE/Visible) CL images of ZnO free-standing nanorod arrays, probed by 3.0 keV e-beam. Each nanorod is homoepitaxially grown from individual growth window with a variety of diameter: (**a**) small diameter growth windows (*D*_w_=100 nm) and (**b**) large diameter growth windows (*D*_w_=500 nm). Nanorods misorientation in *D*_w_=100 nm array takes place during the SEM observation due to electrical charge-up. A bright shadow behind each nanorod array in NBE CL image are signal from ZnO substrate, which is excited due to PMMA thinning by the concentrated e-beam dose. (**c**) Spot-CL spectroscopy at positions 1–8 in **b**, probed by 4.5 keV e-beam. Spectral bands for NBE/Visible CL imaging are highlighted with transparent grey zones. (**d**) A schematic of ZnO free-standing nanorod. Local NBE CL emission efficiency within the nanorod is illustrated by grey scale. The specimen is tilted by 45 degree from ZnO (0001) plane normal. All horizontal and vertical scale bars indicate lengths of 0.5 μm and 1.0 μm, respectively.

Local CL properties are studied for ZnO free-standing nanorods grown homoepitaxially at arrayed circular windows. ZnO nanorod arrays of *D*_w_=100 and 500 nm are investigated by SEM observation and near-band-edge (NBE)/visible (Visible) CL imaging at 3.0 keV from bird's-eye view angles (Fig. 1a,b). Further, local CL spectroscopy is performed at 4.5 keV on a nanorod of *D*_w_=500 nm at spot-CL mode. Eight spot-CL spectra at corresponding e-beam spot positions 1–8 are shown in Fig. 1c, which are correlated by the same colour: position 1 at top *+c* plane, positions 2–7 at side *m* plane from top to the bottom and position 8 on the ZnO *+c* substrate covered with transparent PMMA film. Spectral band of NBE and Visible CL imaging are also indicated by the rectangular grey shadows. Visible CL emission band at 2.1 eV exhibits much more uniform spatial distribution and spectral shape within the nanorod (Supplementary Note 2). Contrary, NBE CL images show that *+c* top-planes are darkest and *m* side-planes gradually become brighter towards the nanorod bottom by one order of magnitude, regardless of *D*_w_. Spot-CL spectroscopy also shows that each NBE CL peak consists of intrinsic emission (3.28 eV) and red-shifted emission (3.19 eV). Intrinsic NBE CL emission is dominant at position 1, however, red-shifted NBE CL emission intensity increases to be dominant and then saturated as the spot position goes from 2 to 7. Considering that SEM e-beam probes CL from ZnO surface-to-depth of Kanaya–Okayama electron range (192 nm for 4.5 keV electron)^59^, red-shifted emission is unique to *m* plane and *m* polar growth thickness seems to be increasing from 0 at the nanorod top-plane to >200 nm at the nanorod bottom. Spatial distribution of NBE CL emission efficiency within a nanorod is illustrated in Fig. 1d, which is further investigated in Figs 3 and 4. Note that each nanorod exhibits some locally bright NBE CL emission spots (CL spots) on *m* side-planes. As spectrum 6 on a CL spot shows the same spectral shape as those at other spot positions on *m* plane, the CL spots have the same properties of *m* plane.

### Low-temperature CL spectroscopy at nanorod top/side plane

**Figure 2:**
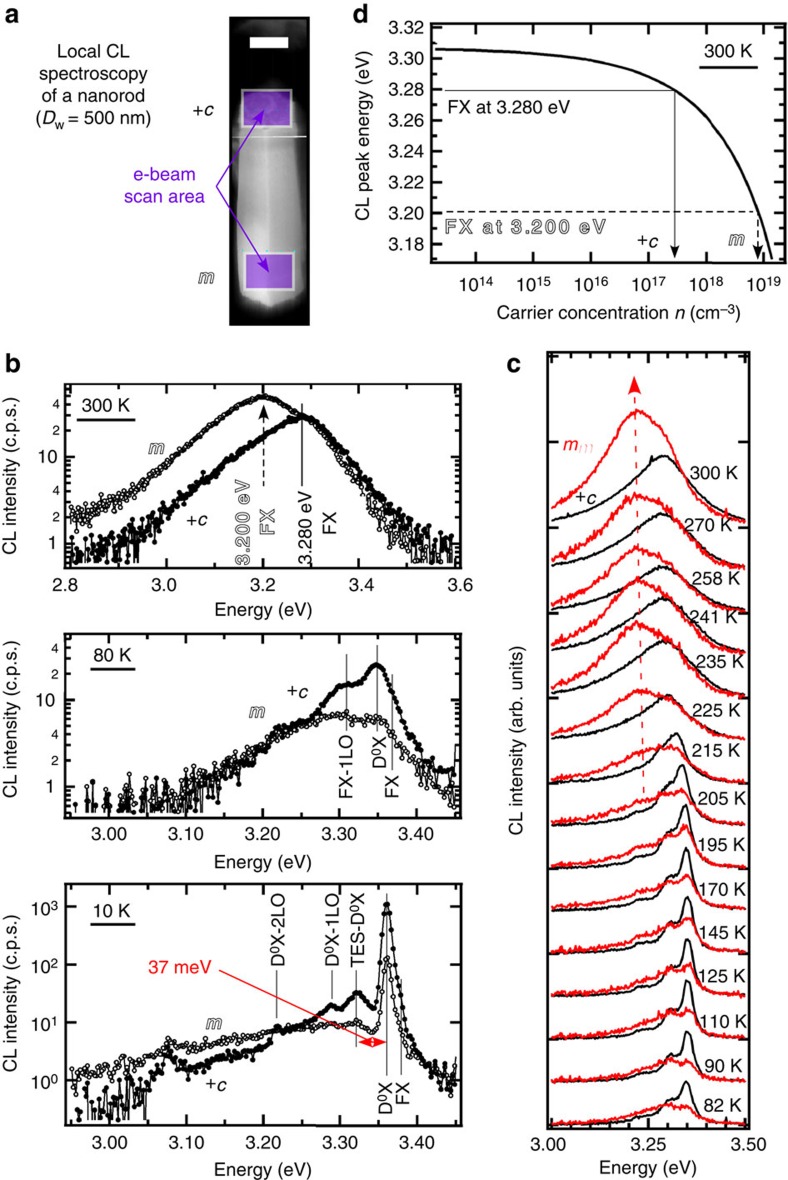
Temperature-dependent high-resolution CL spectroscopy of a nanorod *+c* top-plane and *m* side-plane. (**a**) Bird's-eye view SEM image of a ZnO nanorod investigated by Area-CL spectroscopy. E-beam scan areas on *+c* top-plane and *m* side-plane are indicated by transparent purple boxes. A horizontal scale bar indicates length of 0.5 μm. (**b**) Area-CL spectra on *+c* and *m* planes at 300, 80 and 10 K, probed by 3.0 keV e-beam. CL peaks are assigned to free exciton (FX), neutral donor-bound exciton (D^0^X), its *n*th LO phonon replica (D^0^X-*n*LO) and its two electron satellite (TES-D^0^X). (**c**) Detailed temperature dependence of Area-CL spectra between 80 and 300 K. Emergence of the redshifted FX peak is clearly observed. (**d**) Correlation between FX peak energy *E*_FX_ (eV) and carrier electron concentration *n* (cm^−3^). Residual carrier concentrations *n* of *+c* and *m* growth sectors are evaluated using their correlation: *E*_FX_(*n*)=3.307–8.39 × 10^−15^*n*^2/3^-3.64 × 10^−8^*n*^1/3^ (ref. 61).

To reveal the origin of red-shifted NBE CL emission of *m* plane, temperature-dependent CL spectroscopy is performed on *+c* top-plane and *m* side-plane of a nanorod (*D*_w_=500 nm). A bird's-eye view SEM image of a ZnO free-standing nanorod indicates each e-beam scan area by corresponding solid box superimposed (Fig. 2a). Temperature-dependent 3.0 keV NBE CL spectra are obtained in Area-CL mode at higher wavelength resolution: at 300 K (top), at 80 K (middle) and at 10 K (bottom; Fig. 2b). Each CL peak at 10 K is assigned as follows: 3.37 eV to radiative recombination of free excitons (FX), 3.361 eV to neutral donor-bound excitons (D^0^X) observed in naturally *n*-type ZnO, 3.324 eV to its two electron satellite (TES-D^0^X) and two lower energy peaks with 0.072 eV interspacing to *n*th LO phonon replica of D^0^X peak (D^0^X-*n*LO; *n=*1 and 2). The energy difference between D^0^X (1 s state) and TES-D^0^X (2s and 2p states) peaks of 37 meV equals the three-fourth of donor-binding energy *E*_D_=49 meV in the hydrogenic effective mass approach^60^. At elevated temperatures, D^0^X peaks quench and FX peaks emerge at 80 K and FX peak locates at 3.280 eV on *+c* top-plane and at 3.200 eV on *m* side-plane at 300 K (Fig. 2b). The detailed temperature dependence between 80 and 300 K also reveals the emergence of redshifted FX emission peak above 220 K (Fig. 2c). Such a 80-meV redshift larger than *E*_D_ is not attributed to the donor-binding energy itself. Rather, it is attributed to the band-gap shrinkage or band-tailing of a heavily donor-doped semiconductor observable at elevated temperature, both of which originates in donor ionization and its local perturbation of conduction and valence band-edges.

### Local carrier concentration evaluated by CL nanospectroscopy

Here, room temperature FX peak energy in Fig. 2b is attributed to the local residual carrier concentration *n* (cm^−3^). Room temperature band-edge emission energy of a heavily donor-doped semiconductor redshifts due to its band-gap shrinkage or band-tailing. Giles *et al*. investigated *n*-type ZnO bulk crystals with different carrier concentrations *n* (cm^−3^) by photoluminescence spectroscopy and reported their FX emission energy: *E*_FX_(*n*) (eV)=3.307–8.39 × 10^−15^·*n*^2/3^–3.64 × 10^−8^·*n*^1/3^(solid curve in Fig. 2d)^61^. Based on this work, we obtain *n*_*+c*_=2.8 × 10^17^ cm^−3^ at *+c* top-plane and *n*_*m*_=8.2 × 10^18^ cm^−3^ at *m* side-planes. Carrier concentration gap at 300 K is evidenced qualitatively by FX emission intensity gap between dark *+c* top-plane and bright *m* side-plane, which is intensified by residual carrier concentration.

Also, room temperature NBE CL emission spectroscopy of a ‘ZnO nanocolumn', the nanorod at earlier growth stage, is performed at Spot-CL mode (Supplementary Fig. 3). This nanocolumn also exhibits 30 meV redshift of FX emission at its *m* side-plane. NBE CL emission energy of ZnO nanocolumn is also attributed to the local carrier concentration: 3.28 eV emission to *n*_*+c*_=2 × 10^17^ cm^−3^ in the axial *+c* growth sector and 3.25 eV emission to *n*_*m*_=2 × 10^18^ cm^−3^ in the lateral *m* growth sector. The growth duration-dependent energy redshift also supports our idea that the energy redshift of NBE CL emission is originated from the difference of donor incorporation rates, rather than a certain donor-binding energy.

A quantitative accuracy of *n* values given by CL nanospectroscopy is evaluated in comparison with a net carrier concentration estimated from ‘differential' *I–V* measurements of a ZnO free-standing nanorod (Supplementary Fig. 4). Here, *σ*_e_ is net electrical conductivity in the nanorod. Electron mobility *μ*_e_=1 × 10^1^ cm^2^ V^−1^ s^−1^ in the nanorod is assumed as aqueous solution synthesized ZnO films with Hall mobility of *μ*_e_=12.5 cm^2^ V^−1^ s^−1^ at *n*=6.66 × 10^17^ cm^−3^ are reported^62^. Considering slightly tapered nanorod shape, ‘differential' *I–V* measurements of an individual ZnO free-standing nanorod yields net electrical conductivity, *σ*_e_=0.7 Ω^−1^ cm^−1^. Calculated net residual carrier concentration *n*=*σ*_e_/(*eμ*_e_)=4 × 10^17^ cm^−3^ falls between *n* values of *+c* and *m* growth sectors, which suggests that *n* evaluation by CL nanospectroscopy is quantitative.

### Arbitral cross-section of micro-sampled nanorod made by FIB

**Figure 3:**
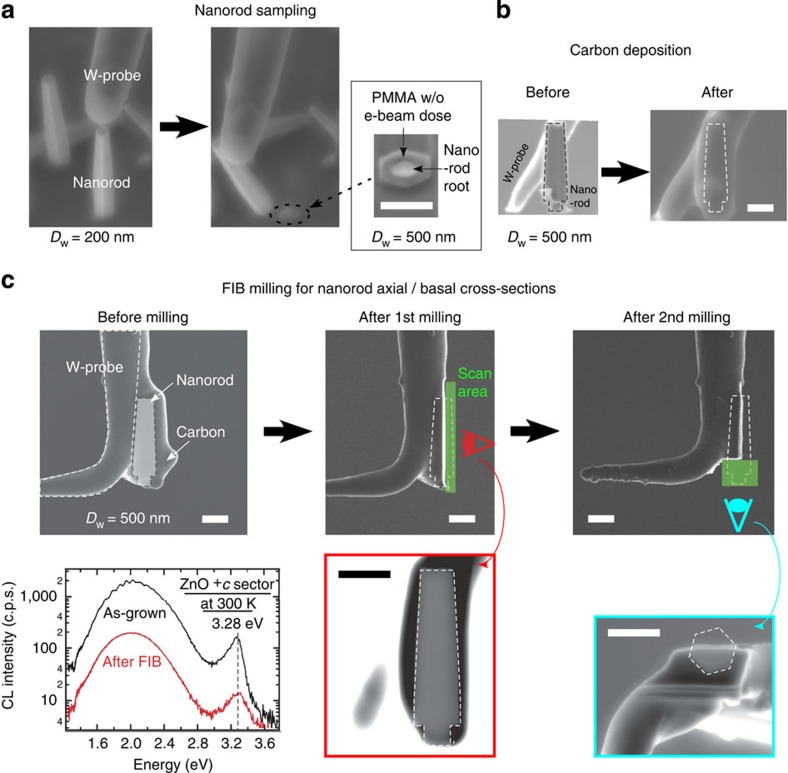
Nanorod micro-sampling and cross-section fabrication by controlling FIB incidence orientation. (**a**) A nanorod (*D*_w_=200 nm) micro-sampling observed by SEM. The inset SEM image shows the circular nanorod root left after the removal of a nanorod (*D*_w_=500 nm). (**b**) SEM images of a micro-sampled nanorod before and after the amorphous carbon deposition. Nanorod position is indicated by dashed lines. (**c**) A series of FIB milling process observed by scanning ion microscope imaging. Nanorod positions and FIB milling scan areas at each step are indicated by dashed line and half-transparent box, respectively. Axial and basal cross-sections fabricated by first and second FIB milling, respectively, are observed consecutively by SEM. The inset at the bottom left shows Spot-CL spectra of the nanorod *+c* top-plane before and after the FIB milling, probed by 4.5 keV e-beam. All scale bars indicate lengths of 1 μm.

Next we show a sequential process to fabricate cross-sections of a micro-sampled free-standing nanorod in arbitral crystallographic orientation. First, a nanorod is micro-sampled using a tungsten (W) probe attached to a nanomanipulator (Fig. 3a). Note that inset SEM image shows a circular root of a nanorod remained attached to the substrate. This root surrounded by PMMA film with a hexagonal white area without e-beam dose, which is originally covered by the nanorod. This micro-sampling reveals circular cylindrical root of the hexagonal nanorod, which suggests that the nanorod growth is limited by PMMA film and that *m* plane growth thickness is evaluated from the diameter gap at the nanorod bottom. The micro-sampled nanorod on the W-probe is then embedded in a carbon film by carbon deposition from different orientations (Fig. 3b).

The W-probe with the nanorod is then transferred into FIB system to fabricate nanorod cross-sections at arbitrary crystallographic orientations by two-step FIB milling (Fig. 3c). Sequential scanning ion microscope images show first FIB milling in axial direction and second FIB milling in basal direction. SEM image of each cross-section observed from eye mark direction is also displayed, which highlights each nanorod cross-section with a bright contrast. Note that the SEM image of second basal cross-section shows that first axial cross-section is fabricated exactly on the nanorod axis, which evidences the high position controllability of this FIB milling technique.

Impact of Ga ion beam milling on surface CL properties is studied by 4.5 keV CL spectra at the nanorod top-plane before and after FIB milling (Fig. 3c inset). After the two-step FIB milling, the CL intensity has dropped to one-tenth due to the residual damaged layer of cross-section surface. Nevertheless, CL spectral shape is retained to investigate local CL properties of the nanorod.

### Growth sectors visualized at arbitrary nanorod cross-section

**Figure 4:**
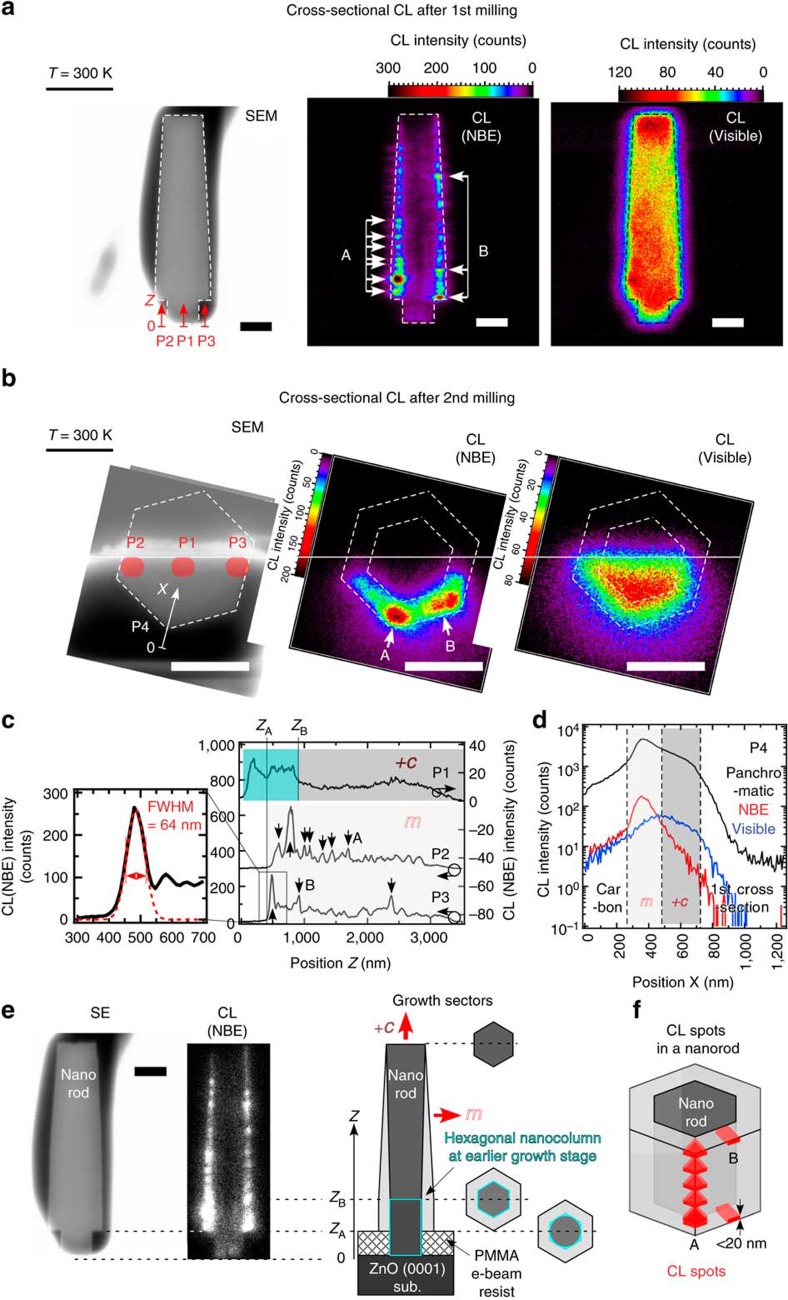
High-resolution cross-sectional CL imaging of a micro-sampled nanorod. SEM and CL (NBE/Visible) images of first axial cross-section (**a**) and those of second basal cross-section (**b**), both of which are probed by 3.0 keV e-beam. Original nanorod volume is indicated by dashed line for eye-guide. (**c**) NBE CL profiles P1–P3 along coordinates *Z* at P1–P3 in **a**. CL spatial resolution is evaluated from FWHM of profile P3 around the sharpest CL spot. (**d**) CL (Panchromatic/NBE/Visible) profiles along coordinate *X*. (**e**) Schematic representation of *+c* and *m* growth sectors within the nanorod cross-section, revealed from comparison between SEM and NBE CL images. Nanorod bottom (*Z*=*Z*_A_) and slight NBE CL intensity drop in *+c* growth sector (*Z*=*Z*_B_) are also indicated in **c**,**e**. CL profiles P1–P4 and a hexagonal nanocolumn at earlier growth stage are indicated by aqua zones and schematically by an aqua box in a *+c* growth sector. All scale bars indicate lengths of 0.5 μm. (**f**) Schematic illustration of spatial distribution and shape of CL spots. Observed CL spots in **a**–**c** are indicated by solid arrows and categorized into either spot A or spot B by their shape and location.

Here we show cross-sectional CL observation of a micro-sampled nanorod to visualize its internal *+c*/*m* growth sectors. Figure 4a,b shows SEM, NBE CL and Visible CL images of first axial cross-section and second basal cross-section at 3.0 kV, respectively. P1–P3 in Fig. 4c are NBE CL line profiles along *Z* axis (// *c*) in Fig. 4a. CL probing regions of P1–P3 are indicated by red areas at nanorod core (centre), nanorod shell (side-plane intersection) and nanorod shell (side-plane centre), respectively, in SEM image in Fig. 4b. Fig. 4d is Panchromatic, NBE and Visible CL profiles along *X* axis in Fig. 4b. Figure 4b,d shows CL quenches near the first axial cross-section because of the damaged surfaces. Two NBE CL images show the two regions with distinct intensity difference: dark hexagonal column core and bright hexagonal shell, whereas two Visible CL images also show them with opposite and weaker contrasts. Note that the bright shell region in NBE CL image corresponds to the diameter gap at the nanorod bottom in SEM image. The diameter gap is formed at an earlier growth stage. A single hexagonal nanocolumn with its diameter equivalent to *D*_w_=0.5 μm is formed by the coalescence of multiple nuclei within each growth window. Subsequently, the lateral growth of the nanocolumn is allowed above PMMA film surface to form a diameter gap (Supplementary Fig. 2b). Thus, we assign the dark core and bright shell in NBE CL images to *+c* and *m* growth sectors of the nanorod, respectively, where each sector has different donor concentration: *n*_*+c*_=2.8 × 10^17^ cm^−3^ in axial *+c* growth sector and *n*_*m*_=8.2 × 10^18^ cm^−3^ in lateral *m* growth sectors, as investigated in Fig. 2. Figure 4e summarizes schematic spatial distribution of *+c* and *m* growth sectors together with corresponding SEM and highlighted NBE CL images.

Also, CL spots are observed in NBE CL images, as indicated by solid arrows and labelled as spot A and B in Fig. 4a,b and as their locations and shapes are illustrated in Fig. 4f. They extend laterally across *m* growth sector and CL spots locate more at the *m* region intersection (spot A) than at their centres (spot B), comparing P2 with P3. A bright CL spot at nanorod bottom is chosen to evaluate the spatial resolution of CL imaging. P3 is locally fitted well with Gaussian curve [*I*(*Z*)=*I*_0_·exp{−(*Z*-*Z*_0_)^2^/2*σ*^2^}] of full-width at half-maximum (FWHM)=2√(2ln2)·*σ=*64 nm. As this FWHM is comparable to Kanaya–Okayama range^59^ of 3.0 keV electron (97 nm), the FWHM is attributed to the lateral resolution of CL imaging.

Note that cross-sectional NBE CL image exhibits two growth turning points, (*Z*_A_, *Z*_B_)=(402 nm, 871 nm), where the axial *+c* growth domain contains a slightly brighter region (0<*Z*<*Z*_B_; Fig. 4c,e). As *Z*_A_ matches with PMMA film thickness (0.3 μm), the turning point at *Z*=*Z*_A_ is attributed to PMMA film surface. Sequential SEM observation of ZnO nanorods at earlier growth stages reveals that the *Z*_B_–*Z*_A_ matches with the typical height (0.5 μm) of each hexagonal nanocolumn above PMMA film (Supplementary Fig. 2b). The turning point at *Z*=*Z*_B_ is then attributed to the formation of single hexagonal nanocolumn (nanorod at earlier stage). CL profiles P1–P4 and a hexagonal nanocolumn at earlier growth stage are indicated by aqua zones and schematically by an aqua box in a *+c* growth sector (Fig. 4c–e). Spot-CL spectroscopy of a nanocolumn reveals NBE CL emission redshift on its side-plane, which evidences that the top-plane and side-planes of the nanocolumn already have distinct difference in their donor incorporation rates (Supplementary Fig. 3b). As nuclei within a growth window also experience axial and lateral growths, the slightly bright region within this nominal +*c* growth sector in Fig. 4e is accountable by the lateral *m* growth sectors of nuclei exposed to the cross-section. Thus, cross-sectional CL imaging of an individual nanorod reveals its growth history from minor contrasts within each growth sector as well as its local carrier concentrations from major contrasts between growth sectors.

## Discussion

The origin of unintentional donors is remained to be discussed. Here we tentatively attribute the residual carriers to interstitial hydrogen donors incorporated from growth environment for the following five reasons: (i) wide-scan X-ray photoemission spectroscopy of reference ZnO homoepitaxial thin film (Supplementary Fig. 5e) does not detect any other possible ZnO donor element than hydrogen, such as Al or Ga, and thus its concentration is typically below 0.1 atomic %; (ii) narrow-scan X-ray photoemission spectroscopy (Supplementary Fig. 5f,g) and Raman spectroscopy (Supplementary Fig. 5h) of the ZnO thin film evidence the presence of hydrogen atoms at bond-centred interstitial sites in the form of [-OH] groups^63^,^64^,^65^,^66^,^67^; (iii) Raman peak observed at 330 cm^−1^ (Supplementary Fig. 5h) is energetically equivalent to the 37 meV gap in 10 K CL peaks in Fig. 2b and thus can be attributed to 1s→2p transitions of interstitial hydrogen donor^66^,^67^,^68^, although it can also be ascribed to second-order vibration mode (E_2_^high^–E_2_^low^) of intrinsic ZnO at 333 cm^−1^ (ref. 69); (iv) D^0^X peak in Fig. 2b matches *I*_4_ peak (3.363 eV) associated with H donor^60^; (v) the *E*_D_=49 meV calculated from CL spectrum at 10 K agrees with *E*_D_=46.1 meV of hydrogen donor reported^60^. Interstitial hydrogen donor in our ZnO nanorod may be originated from Zn(OH)_2_ precursors and incorporated as [-OH] group at oxygen site (Supplementary Note 1). This model is supported by the report on enhanced hydrogen donor incorporation into ZnO (0001) films grown in aqueous solution at higher [OH^−^] concentration (*n*∼10^17^ cm^−3^ at pH=8 and *n*=1.79 × 10^19^ cm^−3^ at pH=10.9)^62^.

Also, we discuss origins of CL spots appeared in *m* growth sectors. The minimum thickness of CL spot origin is at least half an order of magnitude smaller (<20 nm). CL spots extends from the interface of growth sectors along the nanorod basal plane. NBE CL images also show that each CL spot extends laterally, populate preferentially around nanorod corners, and populate more on nanorods of *D*_w_=500 nm than those of *D*_w_=100 nm. Inspired by the above indirect observations, we tentatively consider that CL spots origin might be related with the growth mode of the nanorod side-planes. However, further studies are required to demonstrate this idea.

In summary, we demonstrate that cross-sectional CL technique evaluates local carrier concentration quantitatively at high spatial resolution and at high sensitivity. It also visualizes internal growth sectors of an entire semiconductor nanorod from arbitrary crystallographic orientations, and even reveals nanorod growth history. Our model also gives suggestions how to improve nanorods: (i) nanorod side-plane growth should be minimized as it is the origin of non-uniform diameter and local high carrier concentration; (ii) for aqueous solution growth, amine additives might be promising to enhance uniformities of nanorod diameter and its electrical properties, as they adsorb selectively on nanorod non-polar side-planes and suppress nanorod lateral growth^41^,^42^. Above findings are quite general and are valid for various luminescent semiconductor nanocrystals, regardless of semiconductor species and growth methods.

## Methods

### Selective area homoepitaxy in precursor aqueous solution

In the precursor aqueous solution, ZnO hexagonal nanorods are grown at a time from size-controlled circular holes patterned in PMMA film on an atomically flat ZnO *+c* substrate (selective area homoepitaxy)^53^,^54^. The *c*-axis polarities of these nanorods are expected to be matched considering the recent report on aqueous solution grown ZnO nanowire^70^.

First, Zn-terminated ZnO *+c* substrate was ultrasonically rinsed in acetone, ethanol and deionized water and then annealed at 1,000 °C for 8 h in oxygen atmosphere at 1 atm to make atomically flat surfaces. Then, 300-nm-thick PMMA photoresist is formed on the substrate by spin-coating (Supplementary Fig. 1a). Two hundred circular growth windows per each diameters (*D*_w_=100, 150, 200, 300, 400, 500 nm) are opened in the PMMA film by e-beam lithography to form a 2 × 100 trigonal lattice with 2.0 μm+*D*_w_ interspacing (Supplementary Fig. 1b). Buffered aqueous solution (0.2 l) of equimolar (8 × 10^−3^ M) zinc nitrate hexahydrate (>99.9%, Wako) and hexamethylenetetramine (>99.0%, Wako) without any chemical additive is prepared at room temperature: *T*_aq_=25 °C. The specimen substrate is mounted upside-down in the solution and sealed in the polytetrafluoroethylene (PTFE) container. Then, the container is introduced into the multi-purpose oven set at *T*_set_=85.0 °C and heated for 3.5 h. Therein, ZnO deposition starts at *T*_aq_>60 °C and ZnO nanorods array grow stationary at *T*_aq_=79 °C(Supplementary Note 1). After the heating, the container is cooled naturally down to room temperature and the specimen substrate is removed from the container, rinsed by the deionized water and finally dried by nitrogen gas blow. Contrary to our previous publications^30^,^53^,^54^, PMMA film is not removed from ZnO substrate after the growth.

### SEM-CL observation of a ZnO nanorod

SEM-CL observation is conducted in Nanoprobe-CL system^30^, which is based on a Schottky SEM (Hitachi High-Technologies SU6600). This system equips a SEM specimen cooling stage (Thermal Block Company), which is modified to mount a triaxial piezoelectric nanomanipulator (Kleindiek Nanotechnik MM3A-EM) and triaxial coaxial cables for nano-manipulation and electrical nano-probing. Low-temperature measurements down to 10 K are available by flowing He or N_2_ cooling gas beneath the specimen. SEM-CL nanospectroscopy is performed using e-beam of 3.0 keV and 2.35 nA or that of 4.5 keV and 2.75 nA. E-beam excites CL from ZnO surface to the depth of Kanaya–Okayama range, 97 nm at 3.0 keV and 192 nm at 4.5 keV, respectively^59^. CL is collected by ellipsoidal mirror, dispersed in the spectrometer (Horiba iHR320) and detected by multi-channel charge-coupled device detector (Andor Tech. DU420A-BU2) for CL spectroscopy or by photomultiplier tube (Hamamatsu R943-02) for monochromatic CL imaging. Panchromatic CL analysis is also available using the optical path bypassing the spectrometer. The 300-nm-thick PMMA film on the ZnO substrate is transparent for ZnO CL emission range but plays as an e-beam stopping layer to suppress strong CL from high-quality ZnO substrate. The 3.0-keV CL imaging were performed as short as possible, as e-beam with energy higher than 3.0 keV penetrate PMMA film to excite NBE emission of ZnO substrate and the e-beam dose decompose the PMMA film gradually. CL spectroscopy is performed either in Spot-CL mode at moderate energy resolution (32 meV) and in Area-CL mode at high energy resolution (4.3 meV), where focused e-beam is either spotted or scanned on a nanorod of interest.

### Nanorod micro-sampling and cross-section fabrication by FIB

ZnO nanorods are micro-sampled and their cross-sections are made at desired crystallographic orientations by two-step FIB technique. First, specimens were installed in Nanoprobe-CL system^30^. Single ZnO free-standing nanorod is picked up by electrochemically etched W-probe attached to the nanomanipulator. The probe is then transferred to the carbon coater where carbon film is deposited from various directions to embed the nanorod in a thick amorphous carbon layer. Here, the carbon medium is expected to play several roles: (i) surface protective layer and heat sink from Ga ion beam bombardment; (ii) non-luminescent embedding medium for CL imaging; (iii) embedding medium with appropriate hardness for smooth cross-sections. Each nanorod cross-section is fabricated by two-step FIB milling, coarse milling at 30 keV followed by fine milling at 10 keV, to minimize thickness of damaged layer of the cross-section surface and to improve the S/N ratio of CL imaging.

## Additional information

**How to cite this article:** Watanabe, K. *et al*. Arbitrary cross-section SEM-cathodoluminescence imaging of growth sectors and local carrier concentrations within micro-sampled semiconductor nanorods. *Nat. Commun.* 7:10609 doi: 10.1038/ncomms10609 (2016).

## Supplementary Material

Supplementary Information Supplementary figures 1-5, Supplementary Notes 1-2 and Supplementary References

## References

[b1] Peng X.-G., Wickham J. & Alivisatos A. P. Kinetics of II-VI and III-V colloidal semiconductor nanocrystal growth: ‘Focusing' of size distributions. J. Am. Chem. Soc. 120, 5343–5344 (1998).

[b2] Peng X.-G. . Shape control of CdSe nanocrystals. Nature 404, 59–61 (2000). 10716439 10.1038/35003535

[b3] Nakamura Y., Watanabe K., Fukuzawa Y. & Ichikawa M. Observation of the quantum-confinement effect in individual Ge nanocrystals on oxidized Si substrates using scanning tunneling spectroscopy. Appl. Phys. Lett. 87, 133119 (2005).

[b4] Nakamura Y., Amari S., Naruse N., Mera Y. & Ichikawa M. Self-assembled epitaxial growth of high density beta-FeSi2 nanodots on Si (001) and their spatially resolved optical absorption properties. Cryst. Growth Des. 8, 3019–3023 (2008).

[b5] Li Y. . Dopant-free GaN/AlN/AlGaN radial Nanowire. Nano Lett. 6, 1468–1473 (2006). 16834431 10.1021/nl060849z

[b6] Wang Z.-L. Nanopiezotronics. Adv. Mater. 19, 889–892 (2007).

[b7] Tian B. . Coaxial silicon nanowires as solar cells and nanoelectronic power sources. Nature 449, 885–890 (2007). 17943126 10.1038/nature06181

[b8] Cui Y., Wei Q.-Q., Park H.-K. & Lieber C. M. Nanowire nanosensors for highly sensitive and selective detection of biological and chemical species. Science 293, 1289–1292 (2001). 11509722 10.1126/science.1062711

[b9] Huang M. H. . Room-temperature ultraviolet Nanowire nanolasers. Science 292, 1897–1899 (2001). 11397941 10.1126/science.1060367

[b10] Duan X., Huang Y., Agarwal R. & Lieber C. M. Single-nanowire electrically driven lasers. Nature 421, 241–245 (2003). 12529637 10.1038/nature01353

[b11] Chu S. . Electrically pumped waveguide lasing from ZnO nanowires. Nat. Nanotechnol 6, 506–510 (2011). 21725304 10.1038/nnano.2011.97

[b12] Mariano A. N. & Hanneman R. E. Crystallographic polarity of ZnO crystals. J. Appl. Phys. 34, 384–388 (1963).

[b13] Heiland G. & Kunstmann P. Polar surfaces of zinc oxide crystals. Surf. Sci 13, 72–84 (1969).

[b14] Sekiguchi T., Miyashita S., Obara K., Shishido T. & Sakagami N. Hydrothermal growth of ZnO single crystals and their optical characterization. J. Cryst. Growth 214/215, 72–76 (2000).

[b15] He Y. . Crystal-plane dependence of critical concentration for nucleation on hydrothermal ZnO nanowires. J. Phys. Chem. C 117, 1197–1203 (2013).

[b16] Sakagami N. . Variation of electrical properties on growth sectors of ZnO single crystals. J. Cryst. Growth 229, 98–103 (2001).

[b17] Ballingall J. M. & Wood C. E. C. Crystal orientation dependence of silicon auto compensation in molecular beam epitaxial gallium arsenide. Appl. Phys. Lett. 41, 947–949 (1982).

[b18] Wang W. I., Mendez E. E., Kuan T. S. & Esaki L. Crystal orientation dependence of silicon doping in molecular beam epitaxial AlGaAs/GaAs heterostructures. Appl. Phys. Lett. 47, 826–828 (1985).

[b19] Wulff G. Zur frage der geschwindigkeit des wachstums und der auflosung der krystallflachen. Z. Kristallogr. 34, 449–530 (1901).

[b20] Chernov A. A. The kinetics of the growth forms of crystals. Sov. Phys. Crystallogr 7, 728–730 (1963).

[b21] Borgström L. H. Die geometrische bedingung fur die entstehung von kombinationen. Z. Kristallogr. 62, 1 (1925).

[b22] Alexandru H. V. A macroscopic model for the habit of crystals grown from solutions. J. Cryst. Growth 5, 115–124 (1969).

[b23] Singh M., Verma P., Tung H.-H., Bordawekar S. & Ramkrishna D. Screening crystal morphologies from crystal structure. Cryst. Growth Des. 13, 1390–1396 (2013).

[b24] Dubrovskii V. G., Timofeeva M. A., Tchernycheva M. & Bolshakov A. D. Lateral growth and shape of semiconductor nanowires. Semiconductors 47, 50–57 (2013).

[b25] Storm K. . Spatially resolved Hall effect measurement in a single semiconductor nanowire. Nat. Nanotechnol 7, 718–722 (2012). 23103932 10.1038/nnano.2012.190

[b26] Chen W. . Boron distribution in the core of Si nanowire grown by chemical vapor deposition. J. Appl. Phys. 111, 094909 (2012).

[b27] Chen W. . Incorporation and redistribution of impurities into silicon nanowires during metal-particle-assisted growth. Nat. Commun 5, 4134 (2014). 24920212 10.1038/ncomms5134

[b28] van Benthem K. . Three-dimensional imaging of individual hafnium atoms inside a semiconductor device. Appl. Phys. Lett. 87, 034104 (2005).

[b29] Çelebi C. C. . Surface induced asymmetry of acceptor wave functions. Phys. Rev. Lett. 104, 086404 (2010). 20366956 10.1103/PhysRevLett.104.086404

[b30] Watanabe K. . Band-gap deformation potential and elasticity limit of semiconductor free-standing nanorods characterized *in situ* by scanning electron microscope-cathodoluminescence nanospectroscopy. ACS Nano 9, 2989–3001 (2015). 25689728 10.1021/nn507159u

[b31] Watanabe K., Nakamura Y. & Ichikawa M. Conductive optical-fibre STM probe for local excitation and collection of cathodoluminescence at semiconductor surfaces. Opt. Exp 21, 19261–19268 (2013). 10.1364/OE.21.019261 23938843

[b32] Watanabe K. . Scanning tunneling microscope-cathodoluminescence measurement of the GaAs/AlGaAs heterostructure. J. Vac. Sci. Technol. B 27, 1874–1880 (2009).

[b33] Watanabe K., Nakamura Y. & Ichikawa M. Spatial resolution of imaging contaminations on the GaAs surface by scanning tunneling microscope-cathodoluminescence spectroscopy. Appl. Surf. Sci. 254, 7737–7741 (2008).

[b34] Watanabe K., Nakamura Y. & Ichikawa M. Measurements of local optical properties of Si-doped GaAs (110) surfaces using modulation scanning tunneling microscope cathodoluminescence spectroscopy. J. Vac. Sci. Technol. B 26, 195–200 (2008).

[b35] Mass J. . Cathodoluminescence characterization of hydrothermal ZnO crystals. Superlattices Microstruct. 38, 223–230 (2005).

[b36] Lee W. . Cathodoluminescence study of nonuniformity in hydride vapor phase epitaxy-grown thick GaN films. J. Electron Microscopy 61, 25–30 (2012).

[b37] Lee W. . Cross sectional CL study of the growth and annihilation of pit type defects in HVPE grown (0001) thick GaN. J. Cryst. Growth 351, 83–87 (2012).

[b38] Lee W.-W., Kim S.-B., Yi J., Nichols W. T. & Park W.-I. Surface polarity-dependent cathodoluminescence in hydrothermally grown ZnO hexagonal rods. J. Phys. Chem. C 116, 456–460 (2012).

[b39] Numerical Data and Fundamental Relationships in Science and Technology Vol. 17, of Landolt-Bornstein new series (Springer (1982).

[b40] Christman J. A., Woolcott R. R. Jr., Kingon A. I. & Nemanich R. J. Piezoelectric measurements with atomic force microscopy. Appl. Phys. Lett. 73, 3851–3853 (1998).

[b41] Law M., Greene L. E., Johnson J. C., Saykally R. & Yang P. Nanowire dye-sensitized solar cells. Nat. Mater. 4, 455–459 (2005). 15895100 10.1038/nmat1387

[b42] Zhou Y., Wu W., Hu G., Wu H. & Cui S. Hydrothermal synthesis of ZnO nanorods arrays with the addition of polyethyleneimine. Mater. Res. Bull. 43, 2113–2118 (2008).

[b43] Vayssieres L., Keis K., Lindquist S.-E. & Hagfeldt A. Purpose-built anisotropic metal oxide materials: 3D high oriented microrod array of ZnO. J. Phys. Chem B 105, 3350–3352 (2001).

[b44] Vayssieres L. Growth of arrayed nanorods and nanowires of ZnO from aqueous solutions. Adv. Mater. 15, 464–466 (2001).

[b45] Greece L. E. . Low-temperature wafer-scale production of ZnO Nanowire arrays. Angew. Chem. Int. Ed. 42, 3031–3034 (2003). 10.1002/anie.200351461 12851963

[b46] Tian Z. R., Voigt J. A., Liu J., Mckenzie B. & Mcdermott M. J. Biomimetic arrays of oriented helical ZnO nanorods and columns. J. Am. Chem. Soc. 124, 12954–12955 (2002). 12405815 10.1021/ja0279545

[b47] Govender K., Boyle D. S., Kenway P. B. & O'Brien P. Understanding the factors that govern the deposition and morphology of thin films of ZnO from aqueous solution. J. Mater. Chem. 14, 2575–2591 (2004).

[b48] Sun Y., Riley D. J. & Ashfold M. N. R. Mechanism of ZnO nanotube growth by hydrothermal methods on ZnO film-coated Si substrates. J. Phys. Chem. B 110, 15186–15192 (2006). 16884233 10.1021/jp062299z

[b49] Ashfold M. N. R., Doherty R. P., Ndifor-Angwafor N. G., Riley D. J. & Sun Y. The kinetics of the hydrothermal growth of ZnO nanostructures. Thin Solid Films 515, 8679–8683 (2007).

[b50] Xu S., Lao C., Weintraub B. & Wang Z.-L. Density-controlled growth of aligned ZnO nanowire arrays by seedless chemical approach on smooth surfaces. J. Mater. Res. 23, 2072–2077 (2008).

[b51] Xu S. . Optimizing and improving the growth quality of ZnO nanowire arrays guided by statistical design of experiments. ACS Nano 3, 1803–1812 (2009). 19534470 10.1021/nn900523p

[b52] Xu S. & Wang Z.-L. One-dimensional ZnO nanostructures: solution growth and functional properties. Nano Res 4, 1013–1098 (2011).

[b53] Volk J. . Highly uniform epitaxial ZnO nanorods arrays for nanopiezotronics. Nanoscale Res. Lett. 4, 699–704 (2009). 20596319 10.1007/s11671-009-9302-1 PMC2894249

[b54] Erdérlyi R. . Investigations into the impact of the template layer on ZnO Nanowire arrays made using low temperature wet chemical growth. Cryst. Growth Des. 11, 2515–2519 (2011).

[b55] Pauporté T. h., Lincot D., Viana B. & Pellé F. Toward laser emission of epitaxial nanorods arrays of ZnO grown by electrodeposition. Appl. Phys. Lett. 89, 233112 (2006).

[b56] Belghiti H. E., Pauporté T. & Lincot D. Mechanistic study of ZnO nanorod array electrodeposition. Phys. Stat. Solid. 205, 2360–2364 (2008).

[b57] Könenkamp R. . Thin film semiconductor deposition on free-standing ZnO columns. Appl.Phys. Lett. 77, 2575–2577 (2000).

[b58] Weintraub B., Deng Y. & Wang Z.-L. Position-controlled Seedless growth of ZnO nanorods arrays on a polymer substrate via wet chemical synthesis. J. Phys. Chem. C 111, 10162–10165 (2007).

[b59] Kanaya K. & Okayama S. Penetration and energy-loss theory of electrons in solid targets. J. Phys. D 5, 43–58 (1972).

[b60] Meyer B. K. . Bound exciton and donor-acceptor pair recombinations in ZnO. Phys. Stat. Sol. (b) 241, 231–260 (2004).

[b61] Giles N. C. . Effects of phonon coupling and free carriers on band-edge emission at room temperature in n-type ZnO crystals. Appl. Phys. Lett. 89, 251906 (2006).

[b62] Zhang Y. B., Goh G. K., Ooi K. F. & Tripathy S. Hydrogen-related n-type conductivity in hydrothermally grown epitaxial ZnO films. J. Appl. Phys. 108, 083716 (2010).

[b63] Reynolds J. G. . Shallow acceptor complex in p-type ZnO. Appl. Phys. Lett. 102, 152114 (2013).

[b64] Lavrov E. V., Weber J., Börrnert F., Van der Walle C. G. & Helbig R. Hydrogen-related defects in ZnO studied by infrared absorption spectroscopy. Phys. Rev. B 66, 165205 (2002).

[b65] Lavrov E. V., Börrnert F. & Weber J. Dominant hydrogen-oxygen complex in hydrothermally grown ZnO. Phys. Rev. B 71, 035205 (2005).

[b66] Lavrov E. V., Herklotz F. & Weber J. Identification of two hydrogen donors in ZnO. Phys. Rev. B 79, 165210 (2009). 10.1103/PhysRevLett.102.185502 19518886

[b67] Koch S. G., Lavrov E. V. & Weber J. Interplay between interstitial and substitutional hydrogen donors in ZnO. Phys. Rev. B 89, 235203 (2014).

[b68] Janotti A. & Van der Walle C. G. Fundamentals of zinc oxide as a semiconductor. Rep. Prog. Phys 72, 126501 (2009).

[b69] Cuscó R. . Temperature dependence of Raman scattering in ZnO. Phys. Rev. B 75, 165202 (2007).

[b70] Consonni V. . Selective area growth of well-ordered ZnO nanowire arrays with controllable polarity. ACS Nano 8, 4761–4770 (2014). 24720628 10.1021/nn500620t

