# Supplementary material for: Arbitrary cross-section SEM-cathodoluminescence imaging of growth sectors and local carrier concentrations within micro-sampled semiconductor nanorods
Source: Nat Commun. 2016 Feb 16;7:10609. doi: 10.1038/ncomms10609 (PMC4757765; doi:10.1038/ncomms10609)
Supplement: Supplementary Information — Supplementary figures 1-5, Supplementary Notes 1-2 and Supplementary References [file ncomms10609-s1.pdf]

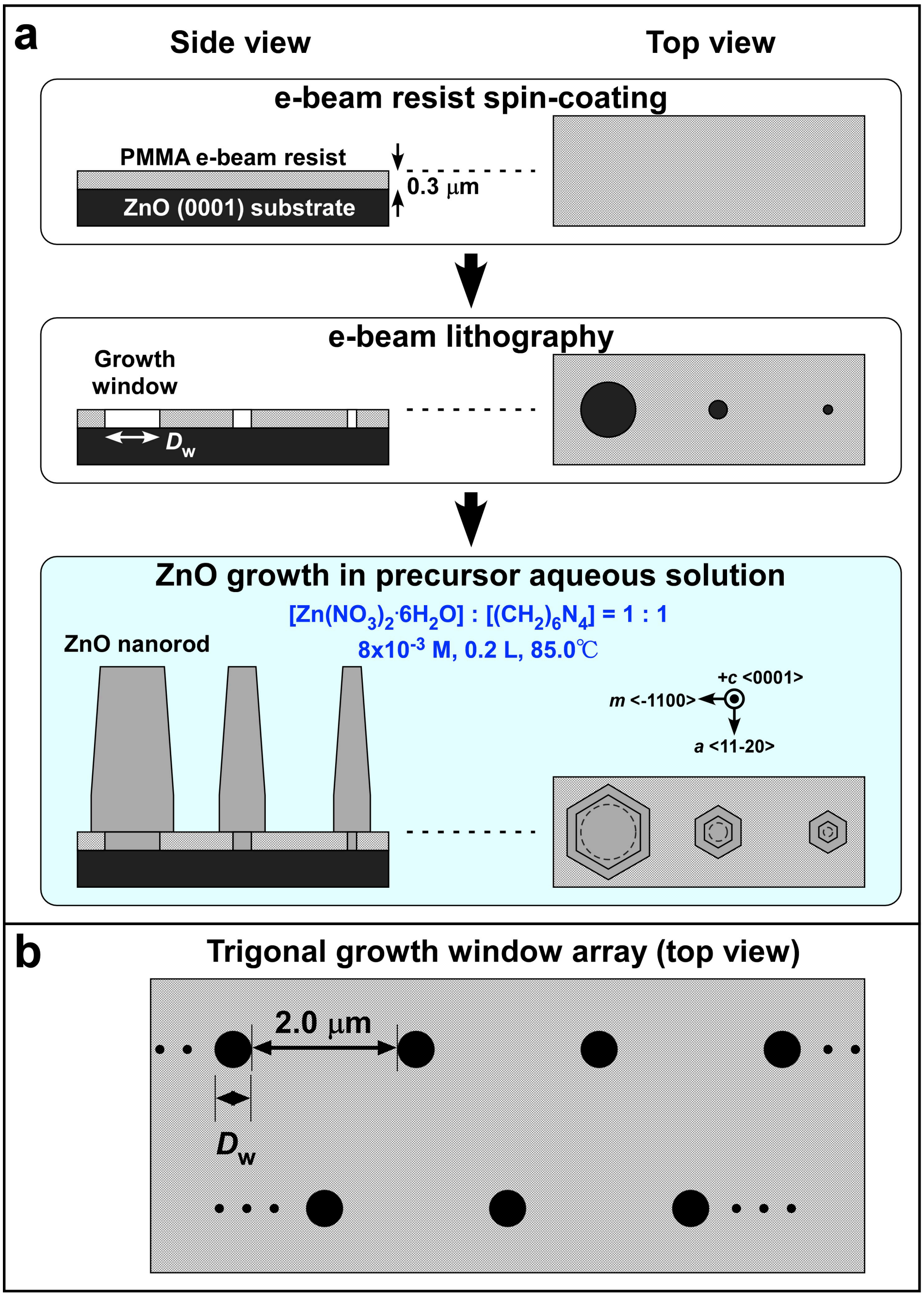

Supplementary Figure 1 | Selective area homoepitaxy of ZnO free-standing nanorod array in precursor aqueous solution. (a) A schematic of selective area homoepitaxy of ZnO free-standing nanorod array in precursor aqueous solution. First, PMMA e-beam resist (0.3 μm-thick) are spin-coated on a ZnO (0001) substrate. Then, circular growth window arrays with different diameters D_w are prepared by electron beam lithography of PMMA e-beam resist film. Finally, the substrate is dipped upside-down in aqueous solution of equimolar zinc nitrate hexahydrate and hexamethylenetetramine (HMTA) precursors (8×10^{-3} M, 0.2 L) and sealed in a container. The container is then introduced in a mechanical oven set at T_{set} = 85.0 ℃, where ZnO nanorod is

grown homoepitaxially at each growth window. (b) A schematic of trigonal circular growth window arrays. The lattice constant is set to 2.0 μm + D_w for each D_w array to minimize the growth interdependency of neighbour windows.

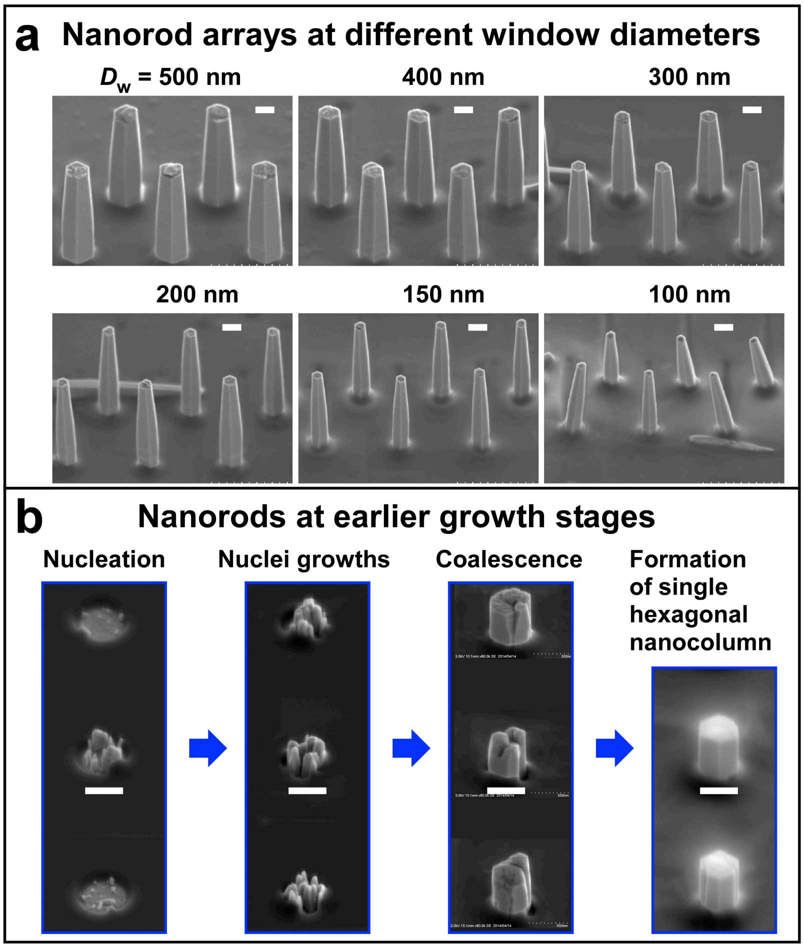

Supplementary Figure 2 | Bird's-eye view SEM observation of ZnO free-standing nanorod arrays. (a) Bird's-eye view SEM images of ZnO free-standing nanorods arrays, which are observed by tilting the ZnO substrate by 45 degree from its (0001):+*c* plane normal toward <11-20>:*a* orientation. Each nanorod has a (0001):+*c* top-plane and six (1-100):*m* side-planes. (b) Sequential bird's-eye view SEM images of ZnO free-standing nanorod array at earlier growth stages (D_w = 500 nm). ZnO nanorod arrays with square lattice constant of 2 μm are grown at identical conditions of precursor aqueous solution and interrupted at different growth durations by quenching the solution. The growth stages within each growth window are categorized into four steps: nucleation, nuclei growths, coalescence of several nuclei, and formation of single hexagonal nanocolumn. A hexagonal nanocolumn typically has a diameter equivalent to D_w = 0.5 μm and a height of 0.5 μm from PMMA film surface. All scale bars indicate lengths of 0.5 μm.

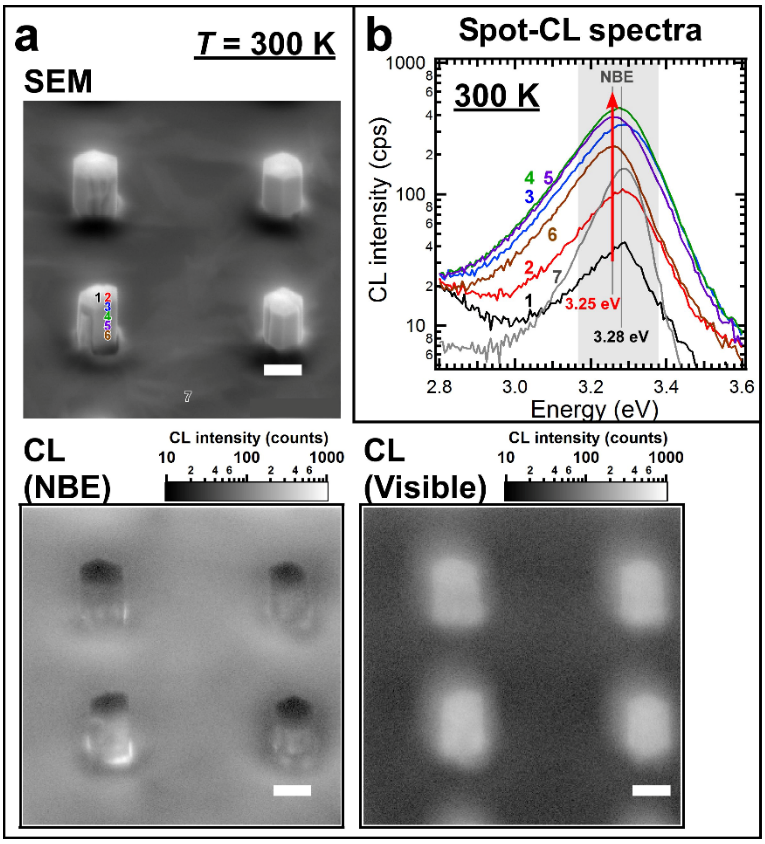

Supplementary Figure 3 |: SEM-CL studies of ZnO free-standing nanorods at earlier growth stage. (a) SEM-CL study (3.0 kV, 2.35 nA) of ZnO free-standing hexagonal nanocolumn array in Supplementary Fig. 2b. Bird's-eye view SEM images are observed in the same manner as those in Supplementary Fig. 2. All scale bars indicate lengths of 0.5 µm. CL images at NBE and Visible emission range show the same trend as Fig. 1. (b) Spot-CL spectra at position 1 (at nanocolumn top–plane), at position 2-6 (at nanocolumn side–plane) and position 7 (on PMMA-covered ZnO (0001) substrate). This nanocolumn also exhibits a redshifted 3.25 eV NBE CL emission at its side-plane as well as the ordinary 3.28 eV NBE CL emission, which becomes dominant as the measurement position goes from 1 to 6. ZnO (0001) substrate exhibits a 3.29 eV NBE CL emission only.

In general, stacking faults or dislocations may be generated by the coalescence of ZnO nuclei which may modulates CL properties locally. However, such minor CL contrasts are not clearly observed. Instead, ZnO nanocolumn exhibits red-shifted NBE CL emission on its side-plane from that on its top-plane, in the same manner as the ZnO nanorod in Fig. 1. This demonstrates that each nucleus grows its +*c* top-plane and *m* side-planes biaxially at different donor incorporation rates. NBE CL emission energy of ZnO nanocolumn is also attributed to the local carrier concentration: 3.28 eV emission to $n_{+c} = 2\times10^{17}$ cm^{-3} in the axial +*c* growth sector and 3.25 eV emission to n_m = 2×10^{18} cm^{-3} in the lateral *m* growth sector.

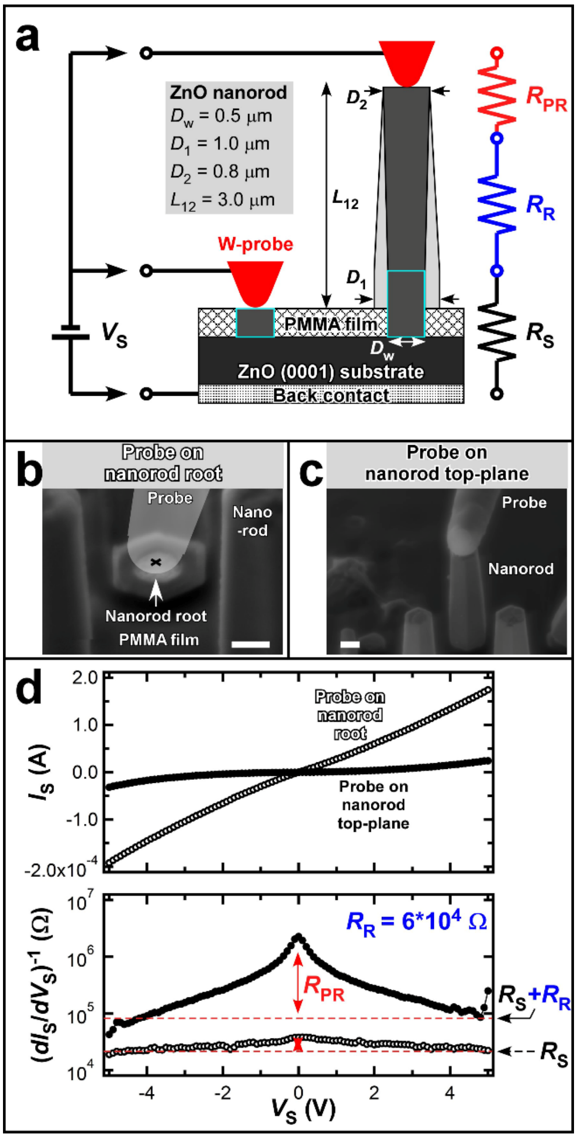

Supplementary Figure 4 | "Differential" *I-V* measurements of an individual ZnO free-standing nanorod. (a) A schematic illustration of the "differential" *I-V* measurement of ZnO free-standing nanorods before or after the nanorod is removed by W-probe micro-manipulation. The ZnO nanorod (D_w = 500 nm) has a height of L_{12} = 3.0 μm and diameters of D_1 = 1.0 μm and D_2 = 0.8 μm. A series resistance between a W-probe and a ZnO (0001) substrate back contact is measured where its equivalent circuit consists of series connections of R_S (resistance between the nanorod root and back contact), R_R (resistance of the nanorod), and R_{PR} (resistance at a W-probe contact on the nanorod top-plane). The R_R of our interest is deduced by subtracting the series resistance when the probe contacts to the nanorod root from that when the probe contacts to the nanorod top-plane. (b)-(c) Corresponding bird's-eye view SEM images observed in the same manner as those in Supplementary Fig. 2. All scale bars indicate lengths of 0.5 μm. The probe contact to the nanorod root is illustrated by the black cross in the half-transparent probe schematic.

Note that the electron beam is blanked during the *I-V* measurements. (d) Comparison of *I-V* curves and resulting differential series resistances with respect to the bias voltage V_S. The differential series resistance in each case decreases with increasing $|V_S|$ and converges at $|V_S| = 5$ V, due to the bias voltage concentration to Schottky W-probe contact and resulting R_{PR} convergence to 0 Ω. Note that the R_{PR} on the nanorod root is lower than R_{PR} on the $+c$ top-plane. This may originates from the hexagonal nanocolumn containing both $+c$ and m growth sectors, which has net carrier concentration higher than pure $+c$ growth sector. The gap between differential series resistances in two cases yields $R_R = 6\times10^4$ Ω. Considering the slightly tapered nanorod shape, net electrical conductivity within a nanorod is estimated to be $\sigma_e = 0.7\ \Omega^{-1}\text{cm}^{-1}$.

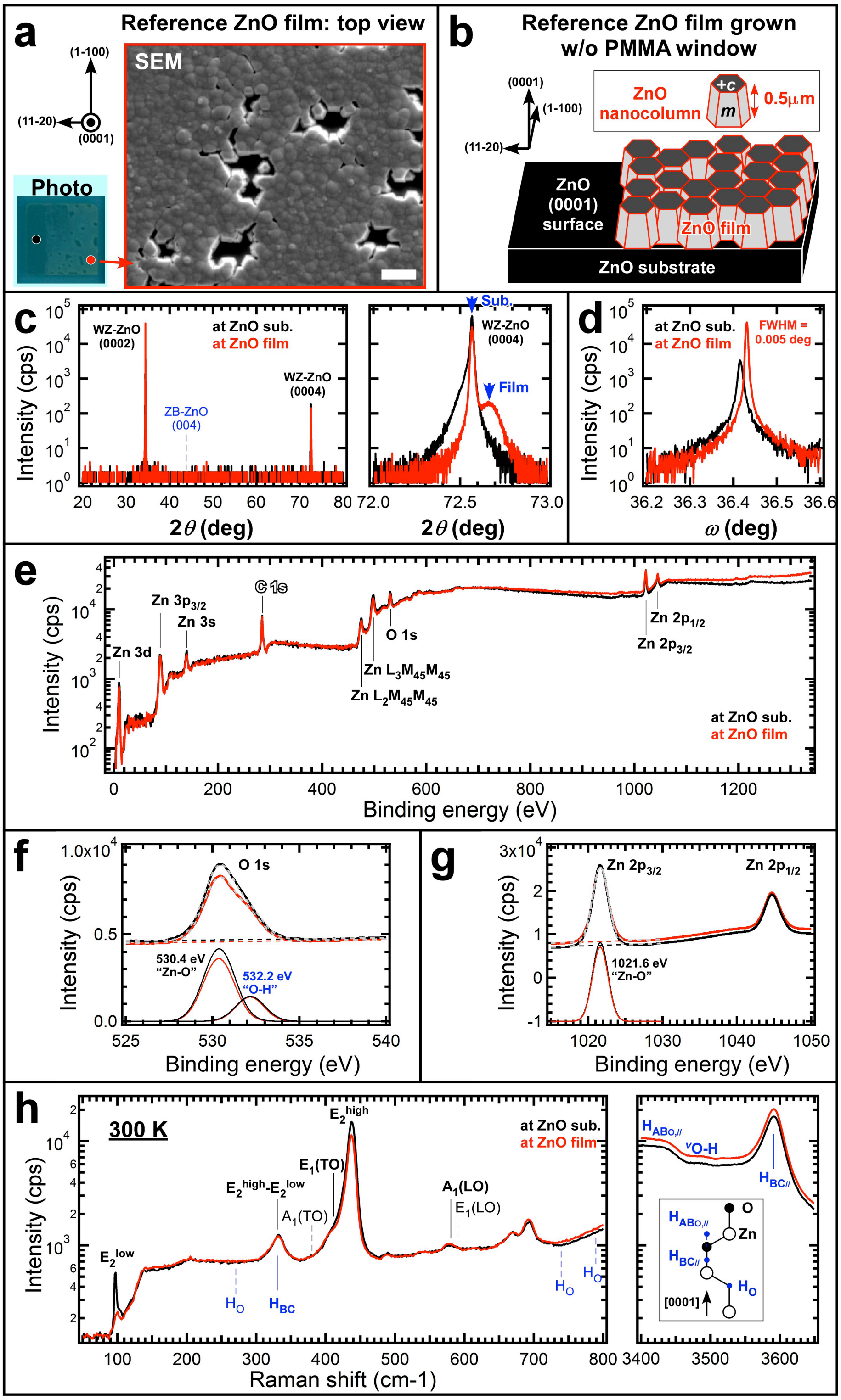

Supplementary Figure 5 | Crystallographic and chemical properties of a reference ZnO thin film. (a) Top view photograph and SEM image of a 0.5 μm-thick reference ZnO thin film which is homoepitaxially grown partially on a hydrothermally synthesized single-crystalline ZnO (0001) substrate ($5\times5\times0.5$ mm^3) at identical conditions of precursor aqueous solution. A scale bar in the

SEM image indicates 0.5 µm in lengths. SEM image reveals that the ZnO thin film is formed by the coalescence of neighbouring ZnO nanocolumns which are distant typically by 150 nm. The photograph also indicates measurement positions at bare substrate and at reference thin film for comparative crystallographic and chemical analyses: x-ray diffraction (XRD) using monochromated Cu $K_{\alpha 1}$ line (λ = 0.15406 nm), x-ray photoelectron spectroscopy (XPS) using monochromated Al K_{α} line (1486.6 eV) with a beam diameter of 0.4 mm, and Raman spectroscopy using a laser diode (λ = 514.5 nm). (b) A schematic of reference ZnO thin film, illustrating that each ZnO nanocolumn grows anisotropically and coalesces to form a film in the same manner as that in Supplementary Fig. 2c. (c) XRD ω-2θ scan curves in wide and narrow ω ranges. Wide scan curve reveals a single-crystalline wurzite (WZ) structure of the film without meta-stable zinc-blende (ZB) phase. Note that narrow scan curve around WZ-ZnO (0004) peak resolves two peaks indicated by blue arrows, where the smaller / larger peak corresponds to the signal from the film / substrate, respectively. Thus, ZnO lattice constant c of the film is slightly (0.14%) smaller than that of the substrate. (d) XRD ω scan curve. FWHM of wurzite ZnO (0004) peaks is 0.005 degree at ZnO film, which is comparable with 0.011 degree at ZnO substrate. Thus, ZnO nanocolumns grown homoepitaxially on the single-crystalline ZnO substrate have small angular dispersions, which then coalesce to form a coherent ZnO film. (e) Wide scan XPS spectra, which demonstrates that impurity elements other than hydrogen are not detected in the reference ZnO thin film and thus their concentrations are typically below 0.1 atomic %. (f-g) Narrow scan XPS O 1s and Zn 2p spectra. Both Zn 2p and O 1s spectra exhibit intrinsic Zn-O bonding peaks, whereas O 1s spectrum also exhibits an extrinsic O-H bonding peak which is more dominant on reference ZnO thin film than on ZnO substrate. (h) Raman spectra. Raman spectra on ZnO thin-film are spatially uniform (convergent within the thickness of red solid line) and significantly different from those on ZnO substrate. Here, visible peaks are indicated by solid lines and thick labels and blind peaks are by dashed lines and thin labels. Raman peaks are identified and labelled with intrinsic ZnO peaks [A_1(LO), E_1(TO), E_2^{high}, E_2^{low}],[1] extrinsic peaks related with impurity hydrogen atoms [H_O, $H_{BC\,//}$, $H_{AB\ O,//}$][2-5] and stretch vibration mode of O-H bond [$^{\nu}$O-H].[6] Inset schematic describes these possible hydrogen atom sites in the ZnO lattice. Raman spectroscopy reveals interstitial hydrogen atoms residing mainly in bond-centred sites of Zn-O bonds orienting to polar c-axis [$H_{BC\,//}$] rather than in anti-bonding sites [$H_{AB\ O,//}$], whereas those in oxygen sites [H_O] are not detected. This suggests a surface-sensitive Raman spectroscopy, probably due to the incident laser light absorption by the mid-gap states of ZnO grown in precursor aqueous solution. The observed 330 cm^{-1} Raman peak can be attributed both to second-order vibration mode[1] [E_2^{high}-E_2^{low}] of intrinsic ZnO observable at 333 cm^{-1} or to transitions between interstitial hydrogen donor states [1s → 2p], since it is equivalent energetically to the 37 meV gap in Fig. 2b.

Supplementary Note 1

ZnO deposition mechanism in HMTA process

Aqueous solution of equimolar zinc nitrate hexahydrate and hexamethylenetetramine (HMTA) precursors (8×10^{-3} M, 0.2 L) is sealed in the container and heated in the multi-purpose oven set at T_{set} = 85.0 ˚C. The solution at 3.5 hours after the heating is measured to be pH ~ 7 by compact pH tester (Hanna Instruments, HI 98127 pHep 4). The ZnO deposition process undergoes by forwarding the intermediate reactions in equilibria. [equations (1)-(3)] Therein, zinc nitrate solution is a weak acid whereas HMTA solution is a weak base. HMTA works as a pH buffer [equations (2)], which realizes stationary growths of ZnO nanorods.[7,8]

$$Zn(NO_3)_2 \cdot 6H_2O \rightarrow Zn^{2+} + 2NO_3^- + 6H_2O \quad (1)$$

$$(CH_2)_6N_4 + 10H_2O \leftrightarrow 6HCHO + 4NH_4^+ + 4OH^- \quad (2)$$

$$Zn^{2+} + 2OH^- \leftrightarrow Zn(OH)_2 \leftrightarrow \text{solid ZnO} + H_2O \quad (3)$$

Reported speciation diagram shows that a large portion of zinc present in solution phase is of the form Zn^{2+} ion. [equation (1)] ZnO precipitates from aqueous solution either directly or via hydroxide. [equation (3)]

Supplementary Note 2

ZnO native point defects and their contributions to Visible CL emission and *n*-type conductivity

Although any ZnO nanorod is homoepitaxially grown single crystals as is revealed by XRD study of referential ZnO homoepitaxial thin film [Supplementary Fig. 5a], it may contain considerable amounts of point defects. ZnO nanorod also exhibits broad Visible CL emission band at 2.1 eV predominantly over NBE CL emission peak at 3.28 eV. [Fig. 1c] Our cross-sectional Visible CL imaging suggests that ZnO native point defects are concentrated more in axial +*c* growth sector than in lateral *m* growth sector, whereas the residual carrier distributions are *vice versa*. Recent studies supports this findings, demonstrating that some of ZnO native point defects such as vacancies (V_{Zn}, V_O), interstitials (Zn_I, O_I), and antisites (Zn_O, O_{Zn}) are associated with Visible CL emission band, however, none of them contribute to the *n*-type conductivity of as-grown ZnO.[9]

Supplementary References.

1. Cuscó R., Alarcón-Lladó E., Ibáñez J., Artús L., Jiménez J., Wang B., Callahan M. J., Temperature dependence of Raman scattering in ZnO. *Phys. Rev. B* **75**, 165202 (2007).

2. Lavrov E. V., Weber J., Börrnert F., Van der Walle C. G., & Helbig R., Hydrogen-related defects in ZnO studied by infrared absorption spectroscopy. *Phys. Rev. B* **66**, 165205 (2002).

3. Lavrov E. V., Börrnert F., & Weber J., Dominant hydrogen-oxygen complex in hydrothermally grown ZnO. *Phys. Rev. B* **71**, 035205 (2005).

4. Lavrov E. V., Herklotz F., & Weber J., Identification of two hydrogen donors in ZnO. *Phys. Rev. B* **79**, 165210 (2009).

5. Koch S. G., Lavrov E. V., & Weber J., Interplay between interstitial and substitutional hydrogen donors in ZnO. *Phys. Rev. B* **89**, 235203 (2014).

6. Reynolds J. G., Reynolds C. L., Jr., Mohanta A., Muth J. F., Rowe J. E., Everitt H. O., & Aspnes D. E., Shallow acceptor complex in p-type ZnO. Appl. Phys. Lett. **102**, 152114 (2013).

7. Xu S. & Wang Z.-L., One-dimensional ZnO nanostructures: solution growth and functional properties. *Nano Res.* 4, 1013-1098 (2011).

8. Ashfold M. N. R., Doherty R. P., Ndifor-Angwafor N. G., Riley D. J., & Sun Y., The kinetics of the hydrothermal growth of ZnO nanostructures. *Thin Solid Films* **515**, 8679-8683 (2007).

9. Janotti A. & Van der Walle C. G., Fundamentals of zinc oxide as a semiconductor. *Rep. Prog. Phys.* **72**, 126501 (2009).
